# Prospects for Food Fermentation in South-East Asia, Topics From the Tropical Fermentation and Biotechnology Network at the End of the AsiFood Erasmus+Project

**DOI:** 10.3389/fmicb.2018.02278

**Published:** 2018-10-15

**Authors:** Yves Waché, Thuy-Le Do, Thi-Bao-Hoa Do, Thi-Yen Do, Maxime Haure, Phu-Ha Ho, Anil Kumar Anal, Van-Viet-Man Le, Wen-Jun Li, Hélène Licandro, Da Lorn, Mai-Huong Ly-Chatain, Sokny Ly, Warapa Mahakarnchanakul, Dinh-Vuong Mai, Hasika Mith, Dzung-Hoang Nguyen, Thi-Kim-Chi Nguyen, Thi-Minh-Tu Nguyen, Thi-Thanh-Thuy Nguyen, Thi-Viet-Anh Nguyen, Hai-Vu Pham, Tuan-Anh Pham, Thanh-Tam Phan, Reasmey Tan, Tien-Nam Tien, Thierry Tran, Sophal Try, Quyet-Tien Phi, Dominique Valentin, Quoc-Bao Vo-Van, Kitiya Vongkamjan, Duc-Chien Vu, Nguyen-Thanh Vu, Son Chu-Ky

**Affiliations:** ^1^Tropical Bioresources & Biotechnology International Joint Laboratory, Université Bourgogne Franche-Comté/AgroSup Dijon- Hanoi University of Science and Technology, Dijon, France; ^2^PAM UMR A 02.102, Université Bourgogne Franche-Comté/AgroSup Dijon, Dijon, France; ^3^Agreenium, Paris, France; ^4^Food Industries Research Institute, Hanoi, Vietnam; ^5^National Institute of Nutrition, Hanoi, Vietnam; ^6^Tropical Bioresources & Biotechnology International Joint Laboratory, Université Bourgogne Franche-Comté/AgroSup Dijon- Hanoi University of Science and Technology, Hanoi, Vietnam; ^7^School of Biotechnology and Food Technology, Hanoi University of Science and Technology, Hanoi, Vietnam; ^8^Atelier du Fruit, Longvic, France; ^9^Food Engineering and Bioprocess Technology, Department of Food, Agriculture and Bioresources, Asian Institute of Technology, Klong Luang, Thailand; ^10^Ho Chi Minh City University of Technology, Ho Chi Minh City, Vietnam; ^11^School of Life Sciences, Sun Yat-sen University, Guangzhou, China; ^12^Institute of Technology of Cambodia, Phnom Penh, Cambodia; ^13^VetoPhage Lyon, France; ^14^Department of Food Science and Technology, Faculty of Agro-Industry, Kasetsart University, Bangkok, Thailand; ^15^Faculty of Food Science and Technology, Vietnam National University of Agriculture, Hanoi, Vietnam; ^16^CESAER, AgroSup Dijon/INRA/Université Bourgogne Franche-Comté, Dijon, France; ^17^Center of Experiment and Practice, Ho Chi Minh City University of Food Industry, Ho Chi Minh City, Vietnam; ^18^International Center for Tropical Agriculture, CGIAR Research Program on Roots, Tubers and Bananas, Cali, Colombia; ^19^Centre de Coopération Internationale en Recherche Agronomique pour le Développement, UMR Qualisud, CGIAR Research Program on Roots, Tubers and Bananas, Montpellier, France; ^20^Institute of Biotechnology, Vietnam Academy of Science and Technology, Hanoi, Vietnam; ^21^Le Centre des Sciences du Goût et de l’Alimentation – AgroSup Dijon/INRA/CNRS/Université Bourgogne Franche-Comté, Dijon, France; ^22^College of Agriculture and Forestry, Hue University, Hue, Vietnam; ^23^Department of Food Technology, Prince of Songkla University, Hat Yai, Thailand

**Keywords:** prospects, food fermentation, South-East Asia, Tropical Fermentation and Biotechnology Network, hurdle technology

## Abstract

Fermentation has been used for centuries to produce food in South-East Asia and some foods of this region are famous in the whole world. However, in the twenty first century, issues like food safety and quality must be addressed in a world changing from local business to globalization. In Western countries, the answer to these questions has been made through hygienisation, generalization of the use of starters, specialization of agriculture and use of long-distance transportation. This may have resulted in a loss in the taste and *typicity* of the products, in an extensive use of antibiotics and other chemicals and eventually, in a loss in the confidence of consumers to the products. The challenges awaiting fermentation in South-East Asia are thus to improve safety and quality in a sustainable system producing tasty and typical fermented products and valorising by-products. At the end of the “AsiFood Erasmus+ project” (www.asifood.org), the goal of this paper is to present and discuss these challenges as addressed by the Tropical Fermentation Network, a group of researchers from universities, research centers and companies in Asia and Europe. This paper presents current actions and prospects on hygienic, environmental, sensorial and nutritional qualities of traditional fermented food including screening of functional bacteria and starters, food safety strategies, research for new antimicrobial compounds, development of more sustainable fermentations and valorisation of by-products. A specificity of this network is also the multidisciplinary approach dealing with microbiology, food, chemical, sensorial, and genetic analyses, biotechnology, food supply chain, consumers and ethnology.

## Introduction

Fermented foods appeared early in the history of mankind. They developed in most cultures as a way to keep food safe and to dress and make tasty everyday dishes. They were produced mainly with spontaneous fermentation, as for instance grape wines or lactic acid fermented vegetables, backslopping, for cheese, and starter inoculation for beer and rice alcohol. With industrialization, an effort was made on pasteurization of raw materials and starter inoculation while the scale of production, previously at the family- or village-scale, increased drastically. This revolution had a real positive impact on the stabilization of product quality and on safety. However, due to the development of world-scale companies, the globalization of tastes, the industrialization of agriculture and the development of transports were promoted, contributing to the pollution of the planet, to a loss of diversity of raw materials and of food products and to the building of a distance between producers and consumers, resulting in a loss of confidence (Ilbery and Kneafsey, 2000; Sonnino, 2007). Moreover, the increase in the scale of production results in an increase in the scale of food safety outbreaks (Pham and Vergote, 2017).

In South-East Asia, despite the developments in agriculture, fermented products are generally still produced at the family- or village-scale with traditional methods. The confidence in safety and quality is however also low and it is necessary to improve the way of production (Pham and Marie-Vivien, 2017). For instance, consumers are not confident in the microbial safety of Nem-chua (fermented sausage) because they are not confident in the microbial safety of locally produced meat (Sarter et al., 2015). The similarly low confidence in vegetables from the local market makes home-producers of fermented vegetables add high contents of salt or vinegar. Even fish sauce (Nuoc-mam), although having being studied for long (Cousin and Noyer, 1944) and sometimes produced at industrial scale, has become recently the center of attention concerning the high levels of histamine.

Between the need to increase quality and safety and the threat of an unsustainable globalizing process leading to a loss of typicity, South-East Asia fermented foods are at the crossroad. Five years ago, researchers from Hanoi University of Science and Technology (HUST) and of AgroSup Dijon/University of Burgundy created a Joint International Laboratory called Tropical Bioresources & Biotechnology to work on the problem of quality, safety, and typicity of South-East Asian fermented foods (Cao-Hoang et al., 2013). In the course of their work, they developed many interactions with other laboratories in Asia and Europe resulting in the creation of an informal network working on tropical fermentation.

The activity of this network was first funded by the French-Speaking Universities Agency (AUF). Now, both in food quality and food safety, the network draws on the current Erasmus+ project called “AsiFood-Universities as key partners for the new challenges regarding food safety and quality in ASEAN”. After an audit of the actual situation in Asia, this capacity building project has created training modules on the various aspects of quality and safety and incorporates them now in Masters and continuous training programs. Among the European partners, Agreenium is the consortium of agriculture, food science and veterinary sciences universities, and research centers. It has a long experience of research in Asia through the CIRAD, AgroSup Dijon, and other members. At the end of this training program, one goal is indeed to strengthens relationships between partners and develop scientific actions toward quality and safety. The Tropical Fermentation Network is thus an axis in the perspectives of AsiFood involving Agreenium, the international lab Tropical Bioresources & Biotechnology and several partners working in fermentation in Vietnam, Cambodia, Thailand, and China.

The strategy of the network to address the problems of quality, safety, typicity, and sustainability is based on a multidisciplinary approach involving economists, for their vision on the organization of the value-chain, food chemists, for the characterization of the products in relation to fermentation, psychologists, to get insights into the role of fermentation for South-East Asian people and study typicity and the perception of sensory properties in the cultural context, and of course, all disciplines of microbiology to investigate ecology, metabolism, physiology, regulation, and biotechnologists to develop sustainable processes. The present manuscript aims at presenting perspectives for the evolution of traditional fermented foods in a context of typicity and sustainability. This view is based on the projects of the Tropical Fermentation Network although only some examples will be given to satisfy the format of the journal.

## Examples of South-East-Asian Fermented Foods

In this paper, we will mention examples dealing with four main fermented foods, lactic acid fermented vegetables and pork (nem-chua), fermented rice and fish sauce (nuoc-mam). Fermented vegetables are usually home- or family-produced products. In urban zones, vegetables are mainly bought in the market without knowledge of origin, they are put in water with salt and the percent of salt is often an indicator of the confidence in the raw-material microbial safety. The product is let for one or several days to let time for a spontaneous fermentation to occur. Nem-chua is the meat equivalent of fermented vegetable. The minced meat mixed with herbs and spices is packed into banana leaves and let for spontaneous fermentation for several days. The main risk concerning nem-chua is related to the meat safety as the product is left uncooked at room temperature.

Nuoc-mam is a kind of fish sauce that can be produced at home but also in industrial companies (Ruddle and Ishige, 2010). It is the result of a long fermentation (often more than 1 year) which is rather unknown. It is one of the Asian food products well known in the world. However, recently, several issues arose. One concerning the quality, despite the importance of Nuoc-mam in Vietnamese cuisine, this product is not protected letting some producers calling Nuoc-mam a mixture of non-fermented materials. The second concern is related to safety and to the detection in some samples of high amount of biogenic amines. A Thai study, which was used by the Codex Alimentarius (Codex 302-2011), pointed out that low amount of fish sauce are eaten and therefore levels of histamine as high as 400 ppm can be tolerated. However, concentrations between 700 and 3000 ppm were detected (Waisundara et al., 2016).

Finally, fermented rice is very popular in many parts of Asia. It can be consumed as a solid or liquid food, be distilled, etc. Traditionally it was prepared from a starter (banh-men in Vietnamese, dombea in Cambodian) containing molds amylolytic- and yeast alcoholic-activities (Vu and Nguyen, 2016). These starters are related to the diversity of products as they are selected from and reused for each local product. Industrial processes changed the amylolytic activities to cooking to reach liquefaction and saccharification resulting in an easier and reproducible process which is also energy consuming. One of the questions is to get something more sustainable and the other is to study the impact of other starter microorganisms on the sensorial properties of the fermented rice (Ly et al., 2018).

## Characterization of the Products and of the Process of Fermentation

As written above, an originality of the project is the multidisciplinary approach to characterize the product and process. This begins with the ethnological dimension of traditional home- and family-scale fermentation. We would like to understand the social representation of fermented food in Vietnam compared to France. Are fermented products more accepted by sensitive-to-local consumers? Or are they conditioned by pure cultural attributes? An ethnographic approach (Valentin and Gomez-Corona, 2018) is used to understand consumer behaviors toward fermented foods. Among the studied points, the choice of raw materials is of importance. Is the origin/state of the raw product a key parameter deciding of the fermentation? Beside the representation of the quality of the product by the consumer, what is the reality concerning the chain of production and supply of raw materials? This study is being carried out in Hanoi and Hue as models of a metropole and a medium-size city. It is possible to have an idea of the amount of vegetables or meat consumed and of those produced locally, imported from another region or from another country. However, when a consumer buys its products at the market, he does not know the origin nor the way followed by the product. These points can be important as the exposure of products to biocides, pollutants, or preservatives can be very dependent on the place of culture (e.g., at the feet of buildings under construction in an expanding city, in the mountain, in delta zones), the level of training of the producers, the number of intermediate people involved. As the production chain is not formally organized and analyses are rare, it is possible that some products undergo several times the same chemical treatment to ensure a nice green color for vegetables, a red one for meat, etc. From the slaughtering capacity and slaughterhouse quality, a good level of microbial safety can be expected from meat produced in Hanoi. However, a recent study showed that for various reasons, the part of livestock slaughtered in modern slaughterhouses is still low (Hoang and Vu, 2017). In this project, we want to make a link between the production and supply chain and the microbial (e.g., presence of pathogens, diversity of lactic acid bacteria, resistance to antibiotics), chemical (presence of biocides/antibiotics, preservatives) and sensorial quality.

These aspects on the origin of raw materials are important for the fermentation, especially in case of spontaneous fermentation, but also for the quality and safety of the product. However, it is still often difficult to characterize the impact of the microbial ecosystem on the product.

### Understanding the Fermentation Ecosystem

The fermenting ecosystem may be very complex but is responsible for a major part of the food typicity. In Western countries, simplification and hygienization concerns drove to the replacement by starters of the microorganisms present in the raw material and in the environment of the workshop and responsible for the spontaneous inoculation. This approach is now incompatible with the European strategy aiming at increasing value through typicity. In South-East Asia, the problem is of improving safety and quality with the use of starters but without losing the taste and product *typicity*! The first microbial step is to characterize the impact of the microorganisms present in the starter on the aroma properties of the product. Fermented rice starters are, due to their link with product typicity, a good model to study. By comparing five traditional starters during fermentation, it was possible to identify correlation between the development of lactic acid bacteria like *Weissella* (W), *Pediococcus* (P), or *Lactobacillus* (L) and the presence of volatile like pentanol (W, P), hexanol (W), ethyl oleate (P), acetic acid (L), ethoxypropanol (L) (Ly et al., 2018). These results are, despite their interest, only preliminary and next studies will have to focus on the interactions between bacteria, yeast and molds and their sensorial impact on the final product. Indeed, the perception of the final product is what matters but evaluating the actions that will improve the quality or the taste of a product is difficult and requires methodological developments of analytical tools focusing on flavor, metabolites and microorganisms (Feng et al., 2014, 2015a,b).

### Finding Technological Starters

To improve the manufacturing and reproducibility of fermented food, starters can be used in addition to the spontaneous ecosystem. With an adequate and controlled addition, the starter can bring quality without impacting the typicity of the product. The selection of starter strains can be made on their capacity to carry out the main fermentation (e.g., production of lactic acid or alcohol), for their metabolic or enzymatic properties (e.g., presence of galactosidases impacting sensorial properties) or for their ability to produce antimicrobial compounds (e.g., bacteriocins). Technological strains can also be used for biotechnological applications as pure cultures. For instance, 84 yeast strains isolated from the fermented sausage-Nem-chua were evaluated for their capacity to produce lactone flavors from castor oil. Five strains of *Yarrowia*, *Moniliella*, and *Candida* sp. were selected for their ability to produce the volatile compound among which, three strains were able to produce more than one g/L in pilote scale conditions (Do et al., 2014). However, to keep the typicity of the product, each function performed by fermentation has to be fulfilled by the starter. Like kefir grains from Caucasus, *banh-men* starter is not a simple mixture of microbial populations but a homeostatic ecosystem. All the microorganisms are developing depending on the complete population even if they have a low impact on the fermentation process (Thanh et al., 2016). From these systems, pure cultures were isolated and mixed to obtain a high performance starter (Dung et al., 2005).

## Food Safety and Food Preservation

The implementation of food preservation technologies is known as a clear need of developing countries (Galvez et al., 2007), which has been confirmed for South-East Asian countries during the AsiFood project. A biopreservation strategy in fermented food can be based either on a hurdle technology, that is, the concept of combining several factors like pH, osmolarity, lactic acid, bacteriocin (Cotter et al., 2005), or on a very active, efficient but specific biocide, like bacteriophages (which can also be used in a hurdle strategy) (Ly-Chatain, 2014). The points that are of importance when building a biopreservation strategy are the risk of promoting resistance (Waché, 2017), of unbalancing the ecosystem or changing its sensorial properties, of poisoning the consumer or the environment and, of course, of having an inefficient system.

The hurdle technology is already applied in traditional fermentation as most recipes include salt, vinegar, some herbs with antimicrobial properties and strains producing antimicrobial compounds. However, for nutritional (decrease the content of salt) and sensorial (avoid the excess of some ingredients, promote a harmonious microbial development) purposes, this step can be improved.

The compounds that can be used as hurdles are products of the metabolism of microorganisms such as organic acids, alcohol, antimicrobial compounds (e.g., bacteriocins), virus active against pathogen bacteria (bacteriophages or phages) and plant extracts. Organic acids and alcohol are part of the composition of the fermented product and cannot be changed but the other compounds are of interest to improve safety of fermented foods.

Bacteriocins are ribosomally synthesized antimicrobial peptides produced by one bacterium that are active against other bacteria, either in the same species (narrow spectrum), or across genera (broad spectrum) (Cotter et al., 2005). In food application, only nisin is accepted as a purified bacteriocin compound but bacterial culture producing bacteriocin *in situ* can be used in fermented food and for biopreservation. Some significant results have already been obtained in fermented vegetables and in the fermented meat *Nem-chua* (Ho, 2017). Several studies have investigated the lactic acid bacteria present in *Nem-chua* and studied the antagonistic functionality of these strains against potential pathogens of pork meat like *Escherichia coli*, *Listeria monocytogenes*, or *Staphylococcus aureus* (Tran and Phan, 2014). It is noteworthy that some bacteria, particularly *Lactobacillus plantarum* strains, produced “broad spectrum” bacteriocins (Tran and Phan, 2014). Among the bacteriocins isolated and further characterized, class II peptides of molecular weight from 14.3 to 20.1 kDa were found in *Lactobacillus plantarum* NCDN4 from *Nem-chua* from Danang province (La Anh, 2015)). Similarly, a 2.5 KDa-class I-peptide was reported to be present in *Lactococcus garvieae* 0–5 isolated from *Nem-chua* obtained from the northern province of Thanh Hoa. In Dua-chua (fermented vegetables) also class II peptides were detected from *Lactobacillus gasseri* (Ho and Wills, 2013). Finally, starter strains of *Lactobacillus plantarum* exhibiting bacteriocin synthesis, protein hydrolysis and some probiotic characteristics were proposed for the production of a “safe nem-chua” (Phan and Chu, 2007; Phan and Nguyen, 2008; Phan, 2011; Phan and Le, 2011). However, the impact of these starters remains to be evaluated on the fermentation and typicity of the product.

Beside bacteriocins many antimicrobial compounds can be used. For instance, plant leaves or their extracts are used in many recipes [e.g., fermented sausage (Nem-chua) and vegetables, rice-starter (Banh-men), etc.] despite a potential antimicrobial effect as discussed for Banh-men by Vu and Nguyen (2016). The plant origin is also an important point. *Litsea cubeba* leaf essential oil from different origin has been shown to exhibit different effects on *E. coli*, showing various targets depending on the plant chemotype (Nguyen et al., 2018). When leaves were used in the diet of common carps, the effect was not only anti *Aeromonas hydrophila* but increased also non specific immunity of the fish (Nguyen et al., 2016). Further studies are thus required to understand the functionality of plant. Recently, a relationship was also made between medicinal plants and the antimicrobial functionality of their ecosystem. Microorganisms from the stem of *Dracaena cochinchinensis* Lour. collected from Cuc Phuong National Park, Ninh Binh province, Vietnam, were screened to find new bioactive compounds (Khieu et al., 2015; Salam et al., 2017). It made possible the isolation of an endophytic strain, *Streptomyces* sp. HUST012, producing a highly potent secondary metabolite active against methicillin-resistant *Staphylococcus* sp., *E. coli*, and *Klebsiella pneumoniae*.

Most of these antimicrobial compounds are not specific and, in fermented food, they can target in the meantime the pathogenic listeria and the starter lactic acid bacteria. There is now much interest on the applications of bacteriophages, which are viruses that can specifically infect bacterial pathogens (Ly-Chatain, 2014). They are very specific, killing only the pathogenic strain targeted. In collaboration with the work of academic partners (Vongkamjan et al., 2017), a research company, VetoPhage^1^, is active in the network, looking for a diversity of phages and working to make them active in feed or veterinary applications. Phages are isolated from farms and food-related environments. Phages with broad-host range ability to lyse bacterial hosts from related sources can be combined as a phage cocktail for controlling in foods- or feed-contaminated-pathogens without leaving by-products like chemical food preservatives or antimicrobials. These systems have a great potential but require careful checking to avoid new bacterial resistance.

Among food safety issues, one important problem is the generation of biogenic amines from amino acids. Biogenic amines are bioactive compounds that can have a role in organism biology. For instance, histamine has the role of a local hormone and neurotransmitter, interacting with cell receptors. They are the product of the decarboxylation of amino acids and are thus potentially present in products rich in hydrolysed proteins and free amino acids. Histamine is thus the decarboxylation product of histidine. Through their interactions with consumers’ cells, biogenic amines present toxicity, observable through various symptoms that can include allergy symptoms like chronic urticaria or headache (Lefèvre et al., 2017).

Decarboxylation may occur readily in acidic conditions or be catalyzed by diamine oxidase bacterial enzymes. Biogenic amine may then be oxidized by amine oxidase. Histamine levels increase rapidly throughout fermentation and reach a peak in the sixth month. After 6 months, the levels of histamine usually diminish, which suggests that prolongation of the fermentation time would allow a significant reduction in histamine content in the final product (Lee et al., 2013). Sanceda et al. (1999) demonstrated that histamine levels decrease during fermentation due to the activity of histamine-degrading bacteria. Strategies based on strains possessing the capacity of oxidizing amines are developed after the observation of correlations between biogenic amines production and microbial species development. However, once again, the dynamic of microbial development has to be better understood with the addition of starter strains.

## Social Approach on Fermented Food as an Advantage for a Safer and Sustainable Supply Chain

### Economic Approach to Understand Roles of Fermentation in the Food System to Identify the Risks While Improving Sustainable Development and Consumer’s Confidence

In developed countries, the conventional food system is characterized by global movements of food and by the domination of intermediary industrial and service operators. The efficient organization of the conventional system has provided easy access to food and a diversity of choice to all consumers. But today, it is criticized for the negative impacts on the environment (the ecological footprint due to intensive agriculture and carbon emissions from transport), on health (undesirable nutritional value), and also in terms of access to food for people with low incomes. The scientific community generally admits that the conventional food system will not be able to assure sustainable development.

For its part, the Vietnamese food system is under the pressure of demography concentration in cities, and is progressively beginning to adopt the European choice, by intensifying production and increasing specialization in different stages of the food chain: production, processing and distribution as an answer. But food model in South-East Asian countries and particularly in Vietnam has not been globalized yet. It represents even certain strengths comparing to the European model. Fermentation could be cheaply developed at households, which is a big advantage in terms of energy consumption. Backed by a local food system, fermentation solution could also help to improve shelf life, decrease losses, and improve food safety.

By combining microbiological research with economic analysis, we study uses of fermentation in food system of Hanoi and Hue City in Vietnam, and postulate that fermentation is an important processing step which contributes to the sustainability of the food chain.

## Making Industrial Fermentation More Sustainable

One direction consists in replacing thermal by biological steps. This strategy is being applied in alcohol production from starch materials. Simultaneous Liquefaction, Saccharification and Fermentation process (SLSF) or no-cook process are developed to enhance ethanol yield and to reduce energy and investment cost (Gohel and Duan, 2012). A mixture of α-amylase and glucoamylase are added to a starch slurry, concomitantly with yeasts, and the SLSF is carried out in a single reactor, at ambient temperature. The simultaneous presence of enzymes and yeast reduces the sugar accumulation in the bioreactor, and because sugar is produced slowly during starch hydrolysis, higher levels of ethanol are reached. Some processes have already been developed from broken rice at very high gravity (VHG) by Chu-Ky et al. (2016) with a raw-starch-hydrolyzing enzyme (mixture of α–amylase and gluco-amylase) protease and active dry-yeast. After 120 h, the SLSF-VHG process reached an ethanol content of 17.6% v/v (86.3% of the theoretical ethanol yield). A similar simultaneous saccharification and fermentation (SSF) process has been developed for cassava flour (Nguyen et al., 2014). In the future, techniques to overcome the drawbacks of VHG condition in beer production like high pitching rate, supplementation of nitrogen and growth factors to wort, application of immobilized yeast, fed-batch fermentation or combined method have to be developed.

## Fermentation as a Tool to Valorise By-Products of the Agriculture and Food Industries

The problem of utilization of by-products is important everywhere but growing environmental concerns in South-East Asia lead to increasing investments in this field (Kumar, 2018). For instance in Vietnam, with the industrialization of the country, a huge amount of by-products has been generated from the agriculture and food industries such as yeast spent, shrimp heads… These resources could be considered co-products as they can generate new products with high added values such as β-glucan, ergosterol (precursor for vitamin D2), hydrolysate of amino acids and chitosan. Dried distillers dried grains (DDG) with solubles were produced from the whole stillage of SLSF process at VHG by being plate-filtered and dried. The obtained DDG had high contents of crude protein (47.5%) and fibers (15.8%). As DDG can still be a rich medium for fungi culture, a project is presently studying its use as a substrate to produce amylases and proteases which could be used for the next rice or cassava liquefaction/saccharification process (Mai et al., personal communication). In Thailand and Vietnam, fermentation of wastewater from several agro-industries (cassava starch, pig, palm oil, etc.) enables a rising production of biogas, which reduces at the same time the environmental impacts of wastewater and the energy costs of the factories using the biogas (Chavalparit and Ongwandee, 2009; Hansupalak et al., 2016).

## Conclusion

**FIGURE 1:**
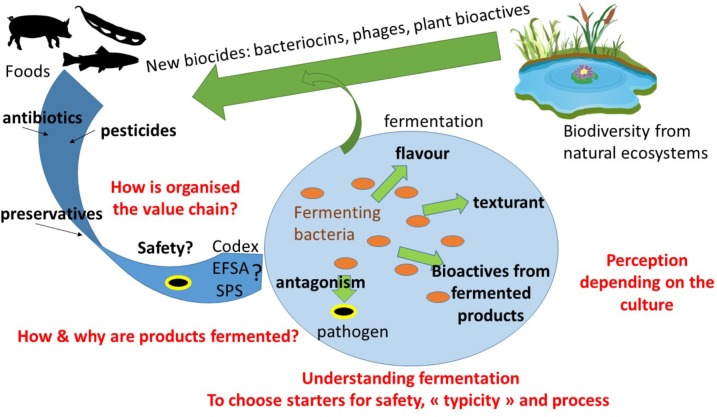
Prospects for food fermentation in South-East Asia with the approach from the Tropical Fermentation and Biotechnology Network.

This paper presents the perspective for the development of fermentation in the coming years in South-East Asia (**Figure 1**). These perspectives are from a network of laboratories. Some strategies can be found in other equivalent countries but some approaches are original as multidisciplinary strategies or as based on specific synergistic collaborations among partners.

## Author Contributions

The choice of the perspective presented is the result of a collective work carried out by all authors. The design and writing of the manuscript is also a collective work involving all authors. YW and SC-K managed the work between the labs and edited the final version to follow the format of the journal.

## Conflict of Interest Statement

The authors declare that the research was conducted in the absence of any commercial or financial relationships that could be construed as a potential conflict of interest.

## Footnotes

1 http://vetophage.fr/
